# Best practice for integrating digital interventions into clinical care for young people at risk of suicide: a Delphi study

**DOI:** 10.1186/s12888-023-05448-7

**Published:** 2024-01-24

**Authors:** Eleanor Bailey, India Bellairs-Walsh, Nicola Reavley, Piers Gooding, Sarah Hetrick, Simon Rice, Alexandra Boland, Jo Robinson

**Affiliations:** 1 Orygen, 35 Poplar Road, Parkville, VIC 3052 Australia; 2 Centre for Youth Mental Health, University of Melbourne, 35 Poplar Road, Parkville, VIC 3052 Australia; 3 Melbourne School of Population and Global Health, University of Melbourne, Grattan Street, Parkville, VIC 3010 Australia; 4 Melbourne Law School, University of Melbourne, Grattan Street, Parkville, VIC 3010 Australia; 5 Faculty of Medical and Health Sciences, University of Auckland, 22-30 Park Ave, Grafton, Auckland, 1023 New Zealand; 6 Present Address: Department of Family Medicine and Community Health, University of Massachusetts Chan Medical School, Worcester, MA USA

**Keywords:** Suicide, Self-harm, Youth, Delphi method, Clinical services, Digital

## Abstract

**Background:**

Digital tools have the capacity to complement and enhance clinical care for young people at risk of suicide. Despite the rapid rise of digital tools, their rate of integration into clinical practice remains low. The poor uptake of digital tools may be in part due to the lack of best-practice guidelines for clinicians and services to safely apply them with this population.

**Methods:**

A Delphi study was conducted to produce a set of best-practice guidelines for clinicians and services on integrating digital tools into clinical care for young people at risk of suicide. First, a questionnaire was developed incorporating action items derived from peer-reviewed and grey literature, and stakeholder interviews with 17 participants. Next, two independent expert panels comprising professionals (academics and clinical staff; n = 20) and young people with lived experience of using digital technology for support with suicidal thoughts and behaviours (n = 29) rated items across two consensus rounds. Items reaching consensus (rated as “essential” or “important” by at least 80% of panel members) at the end of round two were collated into a set of guidelines.

**Results:**

Out of 326 individual items rated by the panels, 188 (57.7%) reached consensus for inclusion in the guidelines. The endorsed items provide guidance on important topics when working with young people, including when and for whom digital tools should be used, how to select a digital tool and identify potentially harmful content, and identifying and managing suicide risk conveyed via digital tools. Several items directed at services (rather than individual clinicians) were also endorsed.

**Conclusions:**

This study offers world-first evidence-informed guidelines for clinicians and services to integrate digital tools into clinical care for young people at risk of suicide. Implementation of the guidelines is an important next step and will hopefully lead to improved uptake of potentially helpful digital tools in clinical practice.

**Supplementary Information:**

The online version contains supplementary material available at 10.1186/s12888-023-05448-7.

## Background

Suicide is a leading cause of death in young people globally [1], and is the number one cause of death in young people in Australia [2]. Suicidal ideation and behaviour (including self-harm and suicide attempt) are more common and associated with risk of future fatal and non-fatal suicide attempts, with international prevalence estimates of suicidal ideation and suicide attempt ranging from 14.3 to 22.6% and 4.6–15.8% respectively [3]. Whilst many young people who experience suicidal thoughts and behaviours do not seek help, many also do; indeed, data indicate that approximately one-third of young people who died by suicide in Australia between 2006 and 2015 were receiving mental health treatment at the time of their death [4]. This indicates a need for suicide prevention efforts to focus on both improving access to services and enhancing the quality of care that services can provide.

Young people are increasingly reliant on digital technologies to seek help and information regarding their mental health, with the COVID-19 pandemic particularly highlighting the potential for telehealth and digital tools to enhance, supplement or extend clinical care [5, 6]. Moreover, an emerging evidence base has demonstrated that digital interventions show promise for the prevention and treatment of suicidal ideation and behaviours in youth [7–9], including when delivered alongside standard clinical care [10]. Despite the emerging evidence base, implementation remains a challenge and the rate of integration of digital interventions into clinical services is low [11, 12]. Several factors are likely at play, including unfamiliarity with available digital interventions and/or how to use them, concerns about the quality or potential utility of available interventions, concerns about how risk of suicide or suicidal behaviour will be assessed, monitored and managed in the digital environment, and time and resource constraints [13–15]. Moreover, no evidence-informed guidance exists for clinicians or services regarding how digital tools can be integrated safely and effectively into clinical care for young people at risk of suicide.

The current study therefore aimed to engage a network of academic experts, mental health professionals, and young people with lived experience to develop a set of best-practice guidelines for integrating digital interventions into clinical care for young people who experience suicidal ideation and/or engage in suicide-related behaviour (including self-harm).

## Methods

### Study design

This study used the Delphi methodology, a method of achieving expert consensus on a particular topic where other study designs are either infeasible or inappropriate, and which offers an opportunity to incorporate practice-based evidence through feedback from expert panellists [16]. The study was conducted over two phases. In the first phase, a questionnaire was developed comprising all possible statements that could go into a set of guidelines. In the second phase, the questionnaire was distributed to two expert panels who rated each of the items according to importance for inclusion in the guidelines. Following two rounds of questionnaires, all items that achieved consensus were compiled into the final guidelines.

### Phase 1: Questionnaire development

Peer-reviewed and grey literature were systematically searched to identify action items. Action items were defined as statements that described what clinicians, service providers, or services (i.e., organisations) should do or had done when using digital tools with young people who experience suicidal thoughts or behaviour.

To identify peer-reviewed articles, Medline, PsycInfo and Embase were searched in July 2020 using the following search string: (suicid* OR self harm OR self-harm) AND (digital OR online OR internet OR technolog* OR ehealth OR e-health OR mhealth OR m-health OR web OR mobile device* OR mobile phone OR cell* phone OR smartphone) AND (young OR youth OR adolescen* OR teen* OR child*). This search strategy identified 1790 records after removing duplicates. Based on title and abstract screening, 165 articles were reviewed in full for eligibility. A total of 52 articles contained relevant action items to extract from and were considered “included”.

To identify grey literature, Google search engines from Australia, New Zealand, Canada, the USA and the UK were searched using four different combinations of search terms related to youth, digital technology, and suicide/self-harm. The first two pages of search results were reviewed. A total of 128 unique grey literature sources were identified via this search strategy, of which 30 contained relevant action items and were considered “included”.

Additionally, due to the paucity of grey or peer-reviewed literature specifically regarding the integration of digital tools into clinical care for this population, qualitative interviews were conducted with key stakeholders with the goal of eliciting additional action items. Two groups of stakeholders were recruited: professionals (n = 9) and consumers (n = 8). Professionals were individuals with clinical or research expertise in digital interventions and youth suicide risk, identified via the research team and contacted via email with an invitation to participate. Inclusion criteria for professional stakeholders were: (1) currently employed in a clinical setting and have experience working with young people with suicidal ideation and/or behaviour (in a client-facing or managerial capacity), (2) have published (or are currently conducting) research evaluating digital interventions for young people at risk of suicide, and (3) living in a predominantly English-speaking country. Consumer stakeholders were recruited via advertisements posted on social media. Inclusion criteria for consumer stakeholders were: (1) aged 15 to 25 inclusive; (2) have used technology for support with suicidal thoughts or behaviour; (3) report experiencing suicidal thoughts “only once or twice” or less in the two weeks prior to consenting as assessed using an adapted version of item 9 of the Patient Health Questionnaire-9 (PHQ-9) [17]. Item 9 of the PHQ-9 was adapted to include the “only once or twice” response (in addition to “not at all”, “several days”, “more than half the days”, and “nearly every day”) to allow participants with fleeting suicidal thoughts to take part. Consumer stakeholders were required to reside in Australia, to enable adequate follow-up in the event of any disclosure of suicide risk during interviews. All interviews were conducted over Zoom, and audio recorded using Zoom’s record function then transcribed. The mean interview length was 34.4 min for professionals and 48.5 min for consumers. Consumer stakeholders were paid $30 per interview (professionals were not paid).

The peer-reviewed and grey literature sources, and qualitative interview transcripts, were then hand-searched for action items. All items meeting criteria were extracted and compiled into an excel spreadsheet. A working group of project team members then reviewed these items in regular meetings. The purpose of the meetings was to review and refine each of the action items, ensuring their clarity and succinctness and that each item conveyed only one idea. Following this process, the action items were compiled into a survey hosted on Qualtrics – the “Round 1” questionnaire.

### Phase 2: Consensus rounds

#### Selection of expert panels

Two expert panels, one of professionals and one of consumers, were recruited. In line with previous studies in this area, the target sample size was 20–30 participants per panel [18, 19]. The inclusion criteria, recruitment processes, and demographic characteristics of the participating panels are described below.

##### Professional panel

Professional panel members were academic experts, clinicians, and leadership/management staff in mental health services.

Academic experts were defined as those who had published (as lead or senior author) at least one peer-reviewed article on the topic of digital interventions for young people who experience suicidal ideation or behaviour. Academic experts were eligible if they were based in any English-speaking country including Australia, New Zealand, Britain, USA and Canada. Academic experts were recruited via contacting first and last authors on all eligible peer-reviewed literature identified via the systematic literature search.

Clinicians were defined as individuals with at least one year of experience providing mental health care to young people who experience suicidal ideation and/or behaviour. Leadership/management staff were defined as individuals with at least one year’s experience overseeing mental healthcare providers who work with young people who experience suicidal thoughts or behaviour. Clinicians and leadership/management staff were only eligible if they were based in Australia or New Zealand and were recruited via advertisements posted to relevant social media groups and on the Australian Psychological Society’s website.

Twenty professional panel members were recruited. Twelve panel members (60.0%) lived in Australia, four (20.0%) in the USA, three (15.0%) in the UK and one (5.0%) in New Zealand. Thirteen identified as female (65.0%) and seven as male (35.0%). The professional panel included three members who also participated in the qualitative interview (Phase 1).

Most professional panel members met eligibility criteria for multiple positions. Eleven (55%) met criteria for “academics”, with years of experience ranging from 1.5 to 31 years (M = 11.1, SD = 8.6). Fifteen (75.0%) met criteria for “clinicians”, with between one- and 40-years’ experience (M = 13.6, SD = 11.5) and eight (40.0%) met criteria for leadership or management staff, with 5–40 years’ experience (M = 15.0, SD = 10.7). Participants worked across a range of settings including universities, non-government and government organisations (including not-for-profits), medical research institutes, and in public and private mental health services.

The twenty professional panel members completed the first questionnaire (Round 1), and nineteen (95.0%) completed the second questionnaire (Round 2). The one panel member who did not complete the second questionnaire did not respond to any emails (i.e., it is unclear why they did not complete Round 2).

##### Consumer panel

Inclusion criteria for consumer panel members were the same as the inclusion criteria for the consumer stakeholder interviewees, described above. Consumer panel members were recruited via social media (with consumer stakeholders from Phase 1 also directly invited to participate). Twenty-nine consumer participants were recruited, including four panel members who also participated in the qualitative interview component (Phase 1). Panel members were asked basic demographic questions, including some questions designed to capture whether the panel was representative of the population of young people known to be at higher risk of suicide. The mean age of panel members was 20.6 (SD = 2.9). Seventeen panel members (58.6%) identified as female, four (13.8%) as male, and eight (27.6%) identified as a gender other than “male” or “female”. Ten panel members (34.5%) described their sexuality as heterosexual; the remaining 19 panel members (65.5%) did not identify this way. Most panel members (n = 22, 75.9%) were born in Australia and most (n = 23, 79.3%) reported English was the main language spoken at home. No panel members identified as Aboriginal or Torres Strait Islander. The majority of consumer panel members lived in Victoria (n = 19, 65.5%), with the remainder living across New South Wales (n = 4, 13.8%), Queensland (n = 4, 13.8%), South Australia (n = 1, 0.3%), and Western Australia (n = 1, 0.3%). Most reported they lived in a metropolitan area (n = 26, 89.7%).

The twenty-nine consumer panel members completed the first questionnaire (Round 1), and twenty-six (89.7%) completed the second questionnaire (Round 2). Of the three who did not complete the questionnaire, one did not respond to any emails and reminders (i.e., it is unclear why they did not complete Round 2), one completed a portion (13.4%) then did not respond to further reminders and thus were removed from the analysis for Round 2, and one had turned 26 years old between the Round 1 and Round 2 questionnaires and advised the research team they did not feel eligible to participate in Round 2.

#### Delphi consensus process

Expert panel members were asked to complete two rounds of questionnaires. In each round, panel members were instructed to rate each item according to its importance for inclusion in the guidelines, using a five-point Likert scale consisting of the following response options: essential, important, unsure/depends, unimportant, should not be included. The items were largely the same for both panels, however a small number of items (n = 49; 19.1%) were removed from the consumer panel survey as they required specific professional expertise (e.g., understanding of service systems); this decision was made in consultation with a youth advisor. At the beginning of both questionnaires, panel members were provided with a list of terminology and definitions which could be downloaded and referenced during questionnaire completion; this included a definition of suicide-related behaviour as including suicide attempt and self-harm regardless of intent. In the first questionnaire (Round 1), all panel members were given the opportunity to suggest additional action items at the end of each section. The second questionnaire (Round 2) included all items that did not reach consensus for either inclusion or exclusion in Round 1, as well as the additional items suggested by panel members.

Consistent with previous Delphi studies developing guidelines [18–20], items were included in the guidelines if they were rated as “essential” or “important” by at least 80% of participants in both panels in Round 1, and were excluded if they were rated as “essential” or “important” by less than 70% of participants in both panels in Round 1. If they were rated as “essential” or “important” by 70–79% of participants in both panels, or if there was a discrepancy between the overall ratings of the two panels (e.g., included by the consumer panel but excluded by the professional panel) in Round 1, these items were re-rated in Round 2. Due to time and resource limitations only two questionnaire rounds were completed, and items not classified as “included” after Round 2 were excluded. In Round 2, panel members were provided with a document containing the Round 1 consensus ratings from both groups for items to be re-rated.

The Round 1 and Round 2 questionnaires were estimated to take 60 and 30 minutes to complete, respectively. Panel members did not have to complete the questionnaire all at once and could save and return to complete it later, providing they accessed it using the same link and device each time. All item responses were forced for the professional panel members, but not for the consumer panel members; this was to allow consumers to skip items that made them feel upset or uncomfortable.

Youth panel members were paid $30 per survey completed. Professional panel members were provided with a $50 AUD-equivalent gift voucher upon completion of the second survey.

## Results

### Consensus ratings

**Fig. 1:**
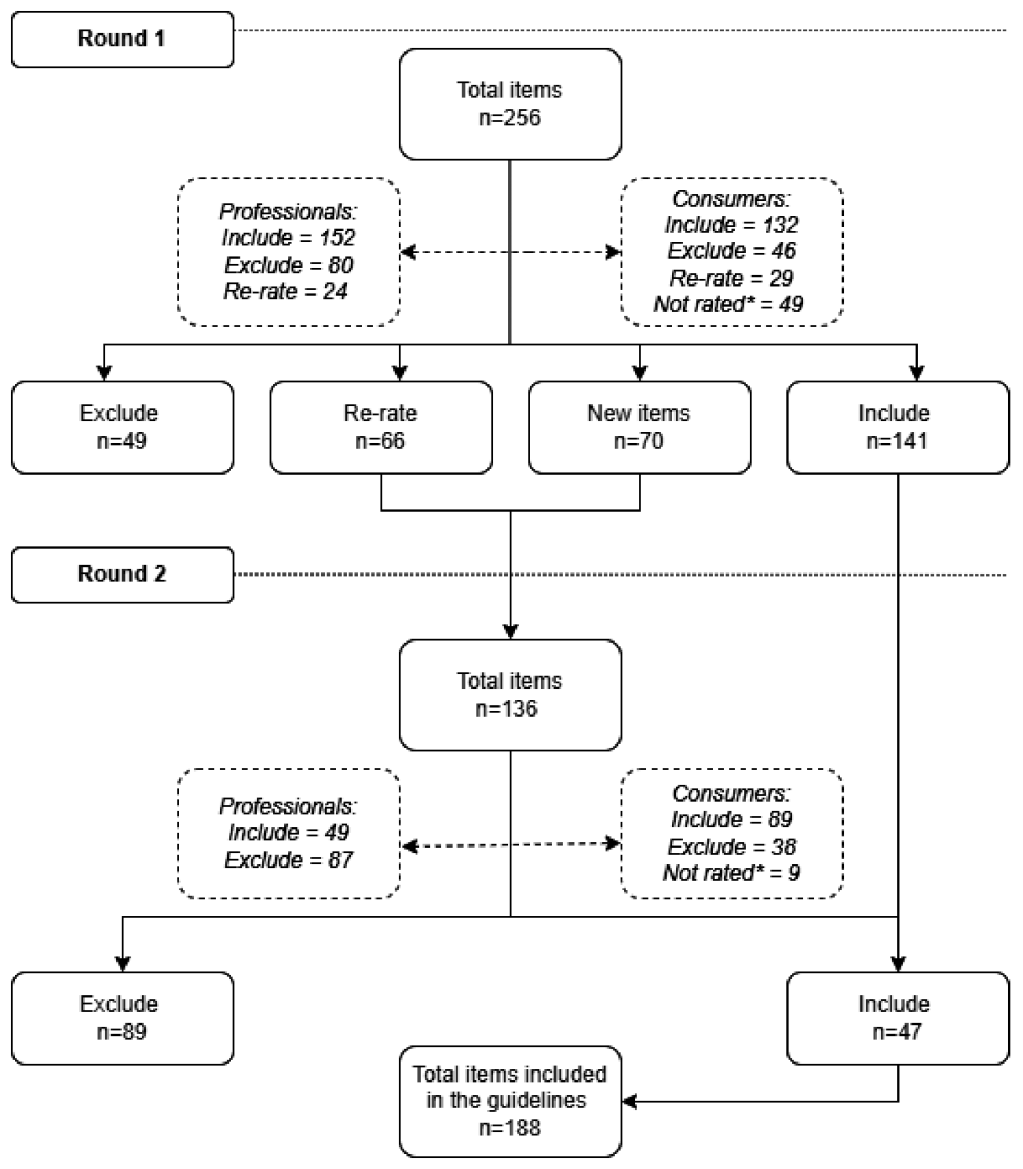
Flow of actions through the Delphi consensus process. *Items not included in the consumer surveys due to requiring professional expertise

Figure 1 displays the flow of action items through the Delphi consensus process. Ultimately, of 326 individual items that were rated (including 70 new items suggested by panel members included in Round 2), 188 (57.7%) were included in the final guidelines [21]. A complete list of every item rated by panel members across both rounds, including their consensus ratings, is contained in Supplementary File 1.

There was strong agreement between the two panels based on combined “essential” and “important” ratings (r = 0.84, p < 0.001). The means and standard deviations of the between-panel differences on combined “essential” and “important” scores were calculated to examine items on which panels disagreed; items with differences of more than two standard deviations above or below the mean are displayed in Table 1. All 14 of these items were rated as “essential” or “important” by a higher proportion of the consumer panel than the professional panel, and two of these had large discrepancies in both questionnaire rounds. Most of the items related to interactive digital tools (e.g., social media, online forums, and chat bots).

**Table 1:** Items where consensus ratings differed significantly between the two panels

	% “essential” or “important”	
Item	Consumers	Professionals
**Questionnaire 1**		
Clinicians should only recommend or endorse online communities to young people who experience suicidal thoughts or behaviour which: clearly display details of when and how often the community is moderated^†^	96.6	60
Clinicians should only recommend or endorse online communities to young people who experience suicidal thoughts or behaviour which: are co-designed with young people with lived experience of suicidal thoughts or behaviour	93.1	55
Clinicians should only recommend or endorse online communities to young people who experience suicidal thoughts or behaviour which: allow users to report posts	100.0	60
*Clinicians should only recommend or endorse online communities to young people who experience suicidal thoughts or behaviour which: allow users to participate using a pseudonym	75.9	30
*As far as possible, clinicians should recommend digital tools which allow users to: input notes from therapy sessions	82.8	45
As far as possible, clinicians should recommend digital tools which allow users to: communicate with other young people who experience suicidal thoughts or behaviour as part of a group	51.7	5
As far as possible, clinicians should recommend digital tools which allow users to: input data about their suicidal thoughts or behaviour “in the moment”	62.1	15
If the clinician attempts to contact the young person to assess their likelihood of engaging in suicidal behaviour, and is not able to reach them, they should: check the young person’s social media profiles to see if they have posted anything that might help with the assessment of risk, only if permission has been obtained by the young person	58.6	20
As far as possible, clinicians should recommend digital tools which have the functionality to: enable direct contact between the young person and their treating mental health clinician (if applicable)	86.2	45
If a young person is considered to be at “high risk of suicide”, the clinician should, at a minimum: recommend digital tool/s which have in-built interactivity features (e.g., a chatbot)	62.1	20
If a young person is considered to be at “acute risk of suicide”, the clinician should, at a minimum: recommend digital tool/s which have in-built interactivity features (e.g., a chatbot)	65.5	20
**Questionnaire 2**		
If a young person starts using a recommended digital tool, and it becomes clear that the tool is used inappropriately by the young person (e.g., used instead of more adaptive help-seeking when in crisis), the clinician should: look for an alternative tool to recommend instead	92.3	47.4
*Clinicians should only recommend or endorse online communities to young people who experience suicidal thoughts or behaviour which: allow users to participate using a pseudonym	84.6	31.6
*As far as possible, clinicians should recommend digital tools which allow users to: input notes from therapy sessions	92.3	36.8

*Large discrepancy (more than two standard deviations above or below the mean) between panels in **both** questionnaires

^†^Reached consensus for inclusion in the guidelines following the Round 2 questionnaire

### Guideline development

At the end of the Round 2 questionnaire, all included items (n = 188) were collated into a set of guidelines [21]. Whilst the wording of the items remained the same, many items were collapsed into single sentences to improve coherence. The structure of the guidelines is shown in Table 2.

**Table 2:** Structure of the guidelines

PART ONE: INTRODUCING DIGITAL TOOLS INTO YOUR CLINICAL PRACTICE
1.1. Developing your knowledge and awareness
1.2. Assessing the young person’s relationship with digital tools and technology
1.3. Selecting a new digital tool for use with a young person
1.4. Recommended features of digital tools
1.5. Online communities
1.6. Explaining a new tool to a young person
1.7. Involvement of parents or carers
PART TWO: IDENTIFYING AND MANAGING RISK OF SUICIDE OR SELF-HARM
2.1. Monitoring the impact of a digital tool on mental state and suicide risk
2.2. Developing standard processes for managing risk
2.3. Developing individualised processes for managing risk
2.4. Responding to risk of suicide or self-harm
2.5. Setting expectations with the young person
2.6. Documentation
PART THREE: ACTIONS FOR SERVICES
3.1. Developing policies and procedures
3.2. Ensuring equity and transparency
3.3. Promoting the uptake of digital tools

## Discussion

This study aimed to develop a set of best-practice guidelines for integrating digital interventions into clinical care for young people who experience suicidal ideation and/or engage in suicide-related behaviour. To our knowledge, these represent the first evidence-informed guidelines on the topic. Forty-nine panel members were recruited, who agreed on the inclusion of 188 items (out of 326; 57.7%). The items provide guidance across three broad areas (divided into three parts in the guidelines): incorporating digital tools into clinical care; identifying and managing risk of suicide; and actions for services. Part One provides guidance on how to choose a new digital tool to introduce to a young person (including the minimum ideal features of digital tools for young people at different levels of suicide risk). This section also advises clinicians to engage young people in ongoing conversations about their use of digital tools (including social media use) and empower young people to recognise the effects of a digital tool on their suicidal thoughts or behaviour and choose digital tools that promote their safety and recovery. In Part Two, clinicians are advised to establish general processes to be followed in the case that a young person indicates via digital means that they may be at risk of suicide or suicidal behaviour, and to develop individualised, specific processes for each young person. For the latter, clinicians are encouraged to include specific indicators of escalating suicidal ideation or behaviour that can be drawn on to assess risk of harm remotely, as well as clear responses to be followed if risk is perceived to be escalating (including who will be contacted in what circumstances). The resulting guidelines do not provide specific advice about exactly what processes should be undertaken based on level of suicide risk, indicating this is a matter of clinical judgment and should be determined on a case-by-case basis. Part Three, “Actions for Services”, includes specific guidance for leadership and/or management in mental health services across three key areas. The first relates to establishing policies and procedures (a “digital strategy”) for the integration of digital tools into the service that stipulate clear governance and risk escalation processes, and the second concerns ensuring equity of access and transparency of processes to young people. The third area provides guidance for promoting the uptake and implementation of digital tools in the service setting, and includes items related to providing training and resources to staff. Taken together, the resulting guidelines are likely to go some way to address several key barriers to uptake of digital tools in clinical practice, including concerns about how to select a digital tool (including how to assess quality or safety), concerns about how to monitor and manage suicide risk in a digital environment, and barriers related to limited knowledge, training, and resourcing [13–15].

A number of items that did not reach consensus for inclusion in the guidelines are worthy of further discussion. For instance, several items related to clinicians looking at a young person’s social media pages (including only with their consent) were included in the survey but did not reach consensus for guideline inclusion. While this does not necessarily indicate the expert panels disagree that clinicians should view young people’s social media pages, it suggests that they do not recommend doing so. Surprisingly, the panels did not agree to include items stipulating that information about means and methods of suicide, images of suicide/self-harm, and content that normalises suicide should be considered “potentially harmful”. This is contrary to recommendations of existing guidelines for reporting on or discussing suicide and suicidal behaviour in traditional media [22] and on social media [23], and suggests contextual factors are important when assessing what content is harmful, and for whom. Indeed, there are likely individual differences in how young people react and respond to such content; moreover, risks may be mitigated if potentially helpful messaging (e.g., that conveys hope or encourages help-seeking) is also present [24, 25]. There was also limited consensus on items related to the use of digital tools by young people who were not in regular contact with a clinician (e.g., post-discharge or on a wait list), suggesting this is likely a nuanced issue which again depends on context. Given digital tools may have great potential to support people as they transition into and out of clinical care [12, 26], and that risk of suicide may be amplified during these periods [27, 28], there is a need for the development of specific guidance in this area.

Items with significant differences in consensus ratings between the two panels were also examined. Interestingly, items stating that clinicians should only recommend online communities that display the details of moderation, are co-designed by young people with lived experience, and allow young people to report posts were all endorsed for inclusion by the consumer panel but were excluded by the professional panel in the Round 1 questionnaire (with all but the first item excluded from the final guidelines). Although the data do not allow examination of the reasons for this, it may be that the professional panel did not support clinicians recommending online communities at all or had feasibility concerns, rather than because they disagreed with the various conditions. A much higher proportion of young people than professionals agreed that young people should be able to participate in online communities using a pseudonym, suggesting that while young people favour the ability to contribute anonymously this is not endorsed by professionals (presumably due to safety concerns, although this could not be confirmed in the current study). Additionally, an item specifying that digital tools should enable direct contact between the young person and their treating clinician was included by the youth panel but excluded by the professional panel, suggesting that while young people would like around-the-clock access to their clinician, this is unlikely to be feasible from the perspective of clinicians. Overall, differences between the ratings of the two panels reflect the differing perspectives of clinicians and consumers: consumers respond based on their lived and living experience, whereas professionals take a more cautious and dispassionate approach. This highlights both the importance of, and challenges associated with, integrating both perspectives in the design and delivery of mental health care.

### Strengths and limitations

This study employed a rigorous Delphi methodology that included a systematic peer-reviewed and grey literature search, stakeholder interviews with groups of young people with lived experience and relevant professionals to supplement gaps in the available literature, and two sufficiently sized panels of topic experts. The use of a consumer panel in addition to a professional panel is a clear strength and aligns with the increasing emphasis on the importance of involving people with lived experience in service design [29]. There was generally strong agreement between the panels and a high completion rate, attesting to the reliability of resulting guidelines. However, the study is not without limitations: due to time constraints, only two consensus rounds were completed. Whilst there is methodological precedent for stopping after two consensus rounds [30], further consensus rounds may have led to the inclusion of more items. We excluded participants who reported frequent suicidal ideation within the week prior to consenting; whilst this was a deliberate safety measure, in consequence the views of this group of young people were not accounted for. Only clinicians and service providers from Australia and New Zealand participated as panel members; as a result, the guidelines specifically target this audience and may have less relevance internationally (particularly in non-English-speaking countries). Despite this, the guidelines provide a foundation for the conduct of further international studies in this area. Finally, it is widely recognised that implementing evidence-based guidelines in healthcare settings is challenging [31], and this study does not address the many barriers to implementing guidelines in practice. To address this, future work by our group will focus on implementing the guidelines through developing accessible learning tools (e.g., webinars, handouts, and templates), developing a strategy to roll out the guidelines and associated tools to the target audience of youth mental health services and clinicians, and evaluating the guideline implementation.

## Conclusions

This study has led to a set of world-first evidence-informed guidelines on integrating digital tools into clinical care for young people who experience suicidal ideation and behaviour. The content of the guidelines has been endorsed by expert professionals and consumers with lived experience, who largely agreed regarding the inclusion of items. It is hoped that the guidelines will address several major barriers to the uptake of digital tools in clinical care, including concerns about the quality of tools and how to assess and manage suicide risk. Whilst these guidelines are an important first step in improving the uptake of potentially efficacious digital tools in clinical care, their existence alone is insufficient; thus, a body of work to facilitate the implementation of the guidelines will be an important next step.

### Electronic supplementary material

Below is the link to the electronic supplementary material.

**Supplementary Material 1:** All items rated by panel members, consensus ratings, and outcome

## Data Availability

The datasets used during the current study are available from the corresponding author upon reasonable request.
