# Supplementary material for: Best practice for integrating digital interventions into clinical care for young people at risk of suicide: a Delphi study
Source: BMC Psychiatry. 2024 Jan 24;24:71. doi: 10.1186/s12888-023-05448-7 (PMC10809499; doi:10.1186/s12888-023-05448-7)
Supplement: Supplementary file 1 — Supplementary Material 1: All items rated by panel members, consensus ratings, and outcome [file 12888_2023_5448_MOESM1_ESM.docx]

**Supplementary File 1: All items rated by panel members, consensus ratings, and outcome**

| **Item** | **Percent rated as “essential” or “important”** | | | | | |
| --- | --- | --- | --- | --- | --- | --- |
|  | **Round 1** | | | **Round 2** | | |
|  | **Consumers** | **Professionals** | **Outcome** | **Consumers** | **Professionals** | **Outcome** |
| Clinicians should make themselves aware of the range of digital tools available for supporting young people who experience suicidal thoughts and behaviour. | 100.0 | 90 | Include |  |  |  |
| For each digital tool being considered for use with young people who experience suicidal thoughts or behaviour, clinicians should ensure that they understand: the quality of the tool (e.g., evidence base, reputation of developers) | 96.6 | 100 | Include |  |  |  |
| For each digital tool being considered for use with young people who experience suicidal thoughts or behaviour, clinicians should ensure that they understand: the possible risks and benefits of using the tool | 100.0 | 95 | Include |  |  |  |
| For each digital tool being considered for use with young people who experience suicidal thoughts or behaviour, clinicians should ensure that they understand: any ethical considerations associated with using the tool (e.g., who has access to data collected via the tool, including in the case of an emergency) | 96.6 | 100 | Include |  |  |  |
| Clinicians should only recommend or use tools which: have been shown to be effective for treating suicidal thoughts or behaviour in a randomised controlled trial (the gold standard method for evaluating interventions) | 82.8 | 85 | Include |  |  |  |
| Clinicians should only recommend or use tools which: have been shown to be safe for young people with suicidal thoughts or behaviour in any interventional research | 96.6 | 95 | Include |  |  |  |
| Clinicians should not recommend digital tools which contain potentially harmful content. | 86.2 | 100 | Include |  |  |  |
| Clinicians should consider the following as "potentially harmful": information about means or methods of suicide or self-harm | 72.4 | 65 | Re-rate | 73.1 | 68.4 | Exclude |
| Clinicians should consider the following as "potentially harmful": images of suicide or self-harm | 79.3 | 65 | Re-rate | 88.5 | 68.4 | Exclude |
| Clinicians should consider the following as "potentially harmful": videos of suicide or self-harm (including live streaming) | 86.2 | 80 | Include |  |  |  |
| Clinicians should consider the following as "potentially harmful": encouragement or promotion of suicide or suicidal behaviour | 89.7 | 80 | Include |  |  |  |
| Clinicians should consider the following as "potentially harmful": content that normalises suicide or suicidal behaviour | 79.3 | 50 | Re-rate | 73.1 | 47.4 | Exclude |
| Clinicians should consider the following as "potentially harmful": content that glamorises or romanticises suicide or suicidal behaviour | 89.3 | 80 | Include |  |  |  |
| Clinicians should consider the following as "potentially harmful": digital tools which allow young people to discuss their experiences of self-harm or suicide with each other | 41.4 | 35 | Exclude |  |  |  |
| Clinicians should consider the following as "potentially harmful": statements of intention to self-harm or suicide (including suicide notes) | 75.9 | 60 | Re-rate | 80.8 | 84.2 | Include |
| Clinicians should not recommend digital tools which allow for social networking between users. | 3.4 | 35 | Exclude |  |  |  |
| Clinicians should be mindful that personal stories about suicide shared via digital tools can be helpful if they use safe language and promote hope and recovery. |  |  | New in Round 2 | 80.8 | 100.0 | Include |
| Clinicians should keep up to date with the relevant literature on the efficacy and effectiveness of digital tools for suicide and suicidal behaviour. | 96.6 | 95 | Include |  |  |  |
| Clinicians should try out digital tools themselves before recommending them to young people (i.e., to ensure they are familiar with how the tool works). |  |  | New in Round 2 | 96.2 | 89.5 | Include |
| Before using digital tools with young people who experience suicidal thoughts or behaviour, clinicians should ensure they have been adequately trained in: assessing and managing suicidal thoughts and behaviour in young people | 100.0 | 95 | Include |  |  |  |
| Before using digital tools with young people who experience suicidal thoughts or behaviour, clinicians should ensure they have been adequately trained in: how to identify potentially harmful content [as rated in Q5 above] | 100.0 | 100 | Include |  |  |  |
| Before using digital tools with young people who experience suicidal thoughts or behaviour, clinicians should ensure they have been adequately trained in the technology behind the tools that they are recommending to the young person (if applicable). |  |  | New in Round 2 | 96.2 | 89.5 | Include |
| In some types of therapy (e.g., aversion therapy), exposure to harmful stimuli (e.g., images of self-harm) is used to induce aversion to those stimuli. If a clinician chooses to use aversion therapy regarding images of self-harm or suicide, exposure to the stimuli should occur in a controlled environment. |  |  | New in Round 2 | 92.3 | 68.4 | Exclude |
| Clinicians should be aware of, and follow, their prevailing code of ethics/practice guidelines when using digital tools with young people who experience suicidal thoughts and behaviour. | 96.6 | 100 | Include |  |  |  |
| Before recommending a digital tool, the clinician should determine: what role they would like the tool to have in monitoring or managing the young person’s suicidal thoughts or behaviour (e.g., does the young person turn to the tool in crisis? Do they use the tool to record when they are experiencing suicidal thoughts? Do they use the tool to communicate with the clinician about suicidal thoughts or behaviour?) | 96.6 | 100 | Include |  |  |  |
| Before recommending a digital tool, the clinician should determine: what outcome they would like the tool to have for the young person (e.g., reduced intensity or frequency of suicidal thoughts, reduced lethality of suicide attempts, reduced incidence of self-harming behaviour, increased coping or problem-solving skills) | 96.6 | 95 | Include |  |  |  |
| If a young person is considered to be at "acute risk of suicide", the clinician should, at a minimum: recommend digital tool/s which have a feature allowing direct and immediate contact with a clinician | 89.7 | 90 | Include |  |  |  |
| If a young person is considered to be at "acute risk of suicide", the clinician should, at a minimum: recommend digital tool/s which have a feature allowing the user to interact with other young people (e.g., an online forum) | 27.6 | 5 | Exclude |  |  |  |
| If a young person is considered to be at "acute risk of suicide", the clinician should, at a minimum: recommend digital tool/s which have a feature allowing direct contact with emergency services | 100.0 | 95 | Include |  |  |  |
| If a young person is considered to be at "acute risk of suicide", the clinician should, at a minimum: recommend digital tool/s which have in-built interactivity features (e.g., a chatbot) | 65.5 | 20 | Exclude |  |  |  |
| If a young person is considered to be at "acute risk of suicide", the clinician should, at a minimum: recommend digital tool/s which Include a safety planning feature | 92.9 | 95 | Include |  |  |  |
| If a young person is considered to be at "acute risk of suicide", the clinician should, at a minimum: not recommend digital tools | 6.9 | 5 | Exclude |  |  |  |
| If a young person is considered to be at "acute risk of suicide", the clinician should, at a minimum: recommend digital tool/s which have a feature allowing the user to interact with other young people (e.g., an online forum), provided appropriate safety measures are in place |  |  | New in Round 2 | 46.2 | 21.1 | Exclude |
| If a young person is considered to be at "acute risk of suicide", the clinician should, at a minimum: recommend digital tool/s which have in-built interactivity features (e.g., a chatbot), provided appropriate safety measures are in place |  |  | New in Round 2 | 69.2 | 42.1 | Exclude |
| If a young person is considered to be at "high risk of suicide", the clinician should, at a minimum: recommend digital tool/s which have a feature allowing direct and immediate contact with a clinician | 96.6 | 90 | Include |  |  |  |
| If a young person is considered to be at "high risk of suicide", the clinician should, at a minimum: recommend digital tool/s which have a feature allowing the user to interact with other young people (e.g., an online forum) | 20.7 | 5 | Exclude |  |  |  |
| If a young person is considered to be at "high risk of suicide", the clinician should, at a minimum: recommend digital tool/s which have a feature allowing direct contact with emergency services | 96.6 | 95 | Include |  |  |  |
| If a young person is considered to be at "high risk of suicide", the clinician should, at a minimum: recommend digital tool/s which have in-built interactivity features (e.g., a chatbot) | 62.1 | 20 | Exclude |  |  |  |
| If a young person is considered to be at "high risk of suicide", the clinician should, at a minimum: recommend digital tool/s which Include a safety planning feature | 72.4 | 85 | Re-rate | 92.3 | 94.7 | Include |
| If a young person is considered to be at "high risk of suicide", the clinician should, at a minimum: not recommend digital tools | 0.0 | 5 | Exclude |  |  |  |
| If a young person is considered to be at "high risk of suicide", the clinician should, at a minimum: recommend digital tool/s which have a feature allowing the user to interact with other young people (e.g., an online forum), provided appropriate safety measures are in place |  |  | New in Round 2 | 46.2 | 5.3 | Exclude |
| If a young person is considered to be at "high risk of suicide", the clinician should, at a minimum: recommend digital tool/s which have in-built interactivity features (e.g., a chatbot), provided appropriate safety measures are in place |  |  | New in Round 2 | 73.1 | 42.1 | Exclude |
| If a young person experiences suicidal thoughts and behaviour but is not considered to be at "high" or "acute risk of suicide", the clinician should, at a minimum: recommend digital tool/s which have a feature allowing direct and immediate contact with a clinician | 69.0 | 55 | Exclude |  |  |  |
| If a young person experiences suicidal thoughts and behaviour but is not considered to be at "high" or "acute risk of suicide", the clinician should, at a minimum: recommend digital tool/s which have a feature allowing the user to interact with other young people (e.g., an online forum) | 55.2 | 20 | Exclude |  |  |  |
| If a young person experiences suicidal thoughts and behaviour but is not considered to be at "high" or "acute risk of suicide", the clinician should, at a minimum: recommend digital tool/s which have a feature allowing direct contact with emergency services | 82.8 | 90 | Include |  |  |  |
| If a young person experiences suicidal thoughts and behaviour but is not considered to be at "high" or "acute risk of suicide", the clinician should, at a minimum: recommend digital tool/s which have in-built interactivity features (e.g., a chatbot) | 51.7 | 20 | Exclude |  |  |  |
| If a young person experiences suicidal thoughts and behaviour but is not considered to be at "high" or "acute risk of suicide", the clinician should, at a minimum: recommend digital tool/s which Include a safety planning feature | 82.8 | 80 | Include |  |  |  |
| If a young person experiences suicidal thoughts and behaviour but is not considered to be at "high" or "acute risk of suicide", the clinician should, at a minimum: not recommend digital tools | 0.0 | 0 | Exclude |  |  |  |
| If a young person experiences suicidal thoughts and behaviour but is not considered to be at "high" or "acute risk of suicide", the clinician should, at a minimum: recommend digital tool/s which have a feature allowing the user to interact with other young people (e.g., an online forum), provided appropriate safety measures are in place |  |  | New in Round 2 | 69.2 | 57.9 | Exclude |
| If a young person experiences suicidal thoughts and behaviour but is not considered to be at "high" or "acute risk of suicide", the clinician should, at a minimum: recommend digital tool/s which have in-built interactivity features (e.g., a chatbot), provided appropriate safety measures are in place |  |  | New in Round 2 | 88.5 | 63.2 | Exclude |
| If a young person is using a digital tool recommended by a clinician or mental health service but is not engaged in face-to-face contact with a clinician (e.g., they are using it while waiting for standard care), a designated clinician should monitor the young person’s suicidal thoughts and behaviour. | 89.7 | 55 | Re-rate | 76.9 | 52.6 | Exclude |
| If a young person is using a digital tool recommended by a clinician or mental health service but is not engaged in face-to-face contact with a clinician (e.g., they are using it while waiting for standard care), AND it becomes evident that their suicidal thoughts or behaviour are escalating, the young person should be offered face-to-face care as soon as possible. | 96.6 | 100 | Include |  |  |  |
| Clinician should recommend digital tools to young people who are not engaged with a clinician in an ongoing way (e.g., young people on waitlist, those who do not meet the services' threshold for intake, or those who have been discharged from the service). |  |  | New in Round 2 | 73.1 | 57.9 | Exclude |
| Clinician should not recommend digital tools to young people who are not engaged with a clinician in an ongoing way (e.g., young people on waitlist, those who do not meet the services' threshold for intake, or those who have been discharged from the service). |  |  | New in Round 2 | 7.7 | 21.1 | Exclude |
| Clinician should recommend digital tools to young people who are not engaged with a clinician in an ongoing way (e.g., young people on waitlist, those who do not meet the services' threshold for intake, or those who have been discharged from the service), but only if: they have a good understanding of the history of the young person |  |  | New in Round 2 | 65.4 | 57.9 | Exclude |
| Clinician should recommend digital tools to young people who are not engaged with a clinician in an ongoing way (e.g., young people on waitlist, those who do not meet the services' threshold for intake, or those who have been discharged from the service), but only if: the young person's safety and wellbeing can be monitored while they use the tool, and any safety issues can be responded to (either by the clinician or others in the service) |  |  | New in Round 2 | 88.5 | 63.2 | Exclude |
| After a young person starts using a recommended digital tool, clinicians should monitor: the impact (positive or negative) of the tool on the young person’s suicidal thoughts or behaviour | 89.7 | 90 | Include |  |  |  |
| After a young person starts using a recommended digital tool, clinicians should monitor: the extent to which the young person complies with any terms agreed upon when the tool was introduced (e.g., whether the tool should be used in a crisis) | 72.4 | 85 | Re-rate | 80.8 | 57.9 | Exclude |
| After a young person starts using a recommended digital tool, clinicians should monitor: any content added to the tool (by the developers or other users) which may be potentially harmful [as rated in Section 1, Q5] | 93.1 | 90 | Include |  |  |  |
| If a young person starts using a recommended digital tool, and it becomes clear that the tool is used inappropriately by the young person (e.g., used instead of more adaptive help-seeking when in crisis), the clinician should: request that the young person stop using the tool | 62.1 | 55 | Exclude |  |  |  |
| If a young person starts using a recommended digital tool, and it becomes clear that the tool is used inappropriately by the young person (e.g., used instead of more adaptive help-seeking when in crisis), the clinician should: look for an alternative tool to recommend instead | 86.2 | 60 | Re-rate | 92.3 | 47.4 | Exclude |
| If a young person starts using a recommended digital tool, and it becomes clear that the tool is potentially harmful for the young person (e.g., is associated with an increase in distress or suicidal ideation), the clinician should: request that the young person stop using the tool | 89.7 | 90 | Include |  |  |  |
| If a young person starts using a recommended digital tool, and it becomes clear that the tool is potentially harmful for the young person (e.g., is associated with an increase in distress or suicidal ideation), the clinician should: look for an alternative tool to recommend instead | 79.3 | 65 | Re-rate | 80.8 | 73.7 | Exclude |
| If a young person starts using a recommended digital tool, and it becomes clear that the tool is used inappropriately by the young person (e.g., used instead of more adaptive help-seeking when in crisis), the clinician should: - New item: offer an alternative that is not digital- or technology-based |  |  | New in Round 2 | 73.1 | 57.9 | Exclude |
| If a young person starts using a recommended digital tool, and it becomes clear that the tool is potentially harmful for the young person (e.g., is associated with an increase in distress or suicidal ideation), the clinician should: - New item: offer an alternative that is not digital- or technology-based |  |  | New in Round 2 | 80.8 | 63.2 | Exclude |
| When determining whether a recommended digital tool is potentially harmful for a young person, clinicians should be guided by the young person’s subjective experience (rather than relying solely on objective measures such as how or when the tool is used). |  |  | New in Round 2 | 92.3 | 68.4 | Exclude |
| Before recommending a digital tool, clinicians should ensure that they understand what is likely to work (or not work) for the individual young person. |  |  | New in Round 2 | 92.3 | 89.5 | Include |
| Clinicians should recommend digital tools based on their knowledge of what is likely to work (or not work) for the individual young person. |  |  | New in Round 2 | 92.3 | 89.5 | Include |
| Clinicians should recommend digital tools based on their knowledge of what is likely to work (or not work) for the individual young person, rather than relying on the broader evidence base. |  |  | New in Round 2 | 57.7 | 42.1 | Exclude |
| Clinicians should be aware of and consider the young person's age and developmental stage when determining which digital tool to recommend. |  |  | New in Round 2 | 88.5 | 100.0 | Include |
| As far as possible, clinicians should recommend digital tools which have been designed in collaboration with young people with lived experience of suicidal thoughts or behaviour. | 96.6 | 80 | Include |  |  |  |
| As far as possible, clinicians should recommend digital tools which Include: a trigger warning upon entry to the digital tool overall | 72.4 | 80 | Re-rate | 76.9 | 68.4 | Exclude |
| As far as possible, clinicians should recommend digital tools which Include: trigger warnings in any specific sections of the tool, as necessary | 86.2 | 80 | Include |  |  |  |
| As far as possible, clinicians should recommend digital tools which Include: direct and clear information about how to access support in a crisis | 100.0 | 100 | Include |  |  |  |
| As far as possible, clinicians should recommend digital tools which Include the following therapeutic features: psychoeducation about suicidal thoughts and behaviour | 82.8 | 90 | Include |  |  |  |
| As far as possible, clinicians should recommend digital tools which Include the following therapeutic features: psychoeducation about mental illness | 86.2 | 65 | Re-rate | 92.3 | 94.7 | Include |
| As far as possible, clinicians should recommend digital tools which Include the following therapeutic features: mood tracking | 89.7 | 70 | Re-rate | 96.2 | 89.5 | Include |
| As far as possible, clinicians should recommend digital tools which Include the following therapeutic features: thought tracking | 89.7 | 70 | Re-rate | 88.5 | 84.2 | Include |
| As far as possible, clinicians should recommend digital tools which Include the following therapeutic features: sleep tracking | 75.9 | 50 | Re-rate | 76.9 | 68.4 | Exclude |
| As far as possible, clinicians should recommend digital tools which Include the following therapeutic features: goal setting | 79.3 | 65 | Re-rate | 76.9 | 73.7 | Exclude |
| As far as possible, clinicians should recommend digital tools which Include the following therapeutic features: emotional regulation strategies | 96.6 | 95 | Include |  |  |  |
| As far as possible, clinicians should recommend digital tools which Include the following therapeutic features: identification of strengths and difficulties | 79.3 | 80 | Re-rate | 80.8 | 73.7 | Exclude |
| As far as possible, clinicians should recommend digital tools which Include the following therapeutic features: identification of personal triggers for experiencing suicidal thoughts or behaviours | 93.1 | 95 | Include |  |  |  |
| As far as possible, clinicians should recommend digital tools which Include the following therapeutic features: monitoring of triggers for suicidal thoughts or behaviours | 89.7 | 95 | Include |  |  |  |
| As far as possible, clinicians should recommend digital tools which Include the following therapeutic features: mindfulness exercises | 79.3 | 50 | Re-rate | 88.5 | 47.4 | Exclude |
| As far as possible, clinicians should recommend digital tools which Include the following therapeutic features: gratitude exercises | 75.9 | 50 | Re-rate | 84.6 | 47.4 | Exclude |
| As far as possible, clinicians should recommend digital tools which Include the following therapeutic features: breathing exercises | 89.7 | 60 | Re-rate | 84.6 | 68.4 | Exclude |
| As far as possible, clinicians should recommend digital tools which Include the following therapeutic features: exercise tutorials | 51.7 | 40 | Exclude |  |  |  |
| As far as possible, clinicians should recommend digital tools which Include the following therapeutic features: yoga exercises | 51.7 | 20 | Exclude |  |  |  |
| As far as possible, clinicians should recommend digital tools which Include the following therapeutic features: a list of strategies the young person can engage in to prevent the onset of suicidal thoughts or behaviour | 96.6 | 100 | Include |  |  |  |
| As far as possible, clinicians should recommend digital tools which Include the following therapeutic features: a list of strategies the young person can implement if they experience suicidal thoughts or feel the urge to engage in suicidal behaviour | 96.6 | 100 | Include |  |  |  |
| As far as possible, clinicians should recommend digital tools which Include the following therapeutic features: a list of strategies to manage suicidal thoughts or behaviour which can be edited or added to by the young person | 96.6 | 95 | Include |  |  |  |
| As far as possible, clinicians should recommend digital tools which Include the following therapeutic features: stories from people with lived experience | 58.6 | 35 | Exclude |  |  |  |
| As far as possible, clinicians should recommend digital tools which Include the following therapeutic features: information/content about suicidal thoughts and behaviour in relation to physical health issues | 75.9 | 40 | Re-rate | 69.2 | 52.6 | Exclude |
| As far as possible, clinicians should recommend digital tools which Include the following therapeutic features: at least one interactive feature | 86.2 | 70 | Re-rate | 84.6 | 57.9 | Exclude |
| As far as possible, clinicians should recommend digital tools which Include the following therapeutic features: recovery-oriented stories from people with lived experience |  |  | New in Round 2 | 80.8 | 52.6 | Exclude |
| As far as possible, clinicians should recommend digital tools which allow users to: input data about their suicidal thoughts or behaviour "in the moment" | 62.1 | 15 | Exclude |  |  |  |
| As far as possible, clinicians should recommend digital tools which allow users to: input their safety or wellness plan | 96.6 | 90 | Include |  |  |  |
| As far as possible, clinicians should recommend digital tools which allow users to: input notes from therapy sessions | 82.8 | 45 | Re-rate | 92.3 | 36.8 | Exclude |
| As far as possible, clinicians should recommend digital tools which allow users to: input images or videos which might form part of their safety plan or coping strategies | 89.7 | 70 | Re-rate | 80.8 | 73.7 | Exclude |
| As far as possible, clinicians should recommend digital tools which allow users to: input pre-written texts which can be quickly distributed to support people if required (e.g., in the case of escalating suicidal ideation) | 93.1 | 80 | Include |  |  |  |
| As far as possible, clinicians should recommend digital tools which allow users to: share information relating to their suicidal thoughts or behaviours with nominated support people (e.g., parents, carers, or friends) | 62.1 | 55 | Exclude |  |  |  |
| As far as possible, clinicians should recommend digital tools which allow users to: share information relating to their suicidal thoughts or behaviours with their treating clinician | 82.8 | 85 | Include |  |  |  |
| As far as possible, clinicians should recommend digital tools which allow users to: share information relating to their suicidal thoughts or behaviours with any health professionals involved in their care | 65.5 | 60 | Exclude |  |  |  |
| As far as possible, clinicians should recommend digital tools which allow users to: choose whether, and what, information relating to their suicidal thoughts or behaviour is shared with others | 75.9 | 75 | Re-rate | 76.9 | 78.9 | Exclude |
| As far as possible, clinicians should recommend digital tools which allow users to: communicate with other young people who experience suicidal thoughts or behaviour one-on-one | 24.1 | 5 | Exclude |  |  |  |
| As far as possible, clinicians should recommend digital tools which allow users to: communicate with other young people who experience suicidal thoughts or behaviour as part of a group | 51.7 | 5 | Exclude |  |  |  |
| As far as possible, clinicians should recommend digital tools which allow users to: allow users to contact emergency services or crisis contacts through the tool | 96.6 | 90 | Include |  |  |  |
| As far as possible, clinicians should recommend digital tools which allow users to: allow users to contact emergency services or crisis contacts with a minimum number of clicks | 89.7 | 95 | Include |  |  |  |
| As far as possible, clinicians should recommend digital tools which allow users to input: contact details for emergency services | 96.6 | 100 | Include |  |  |  |
| As far as possible, clinicians should recommend digital tools which allow users to input: contact details local area mental health services | 93.1 | 100 | Include |  |  |  |
| As far as possible, clinicians should recommend digital tools which allow users to input: the location of their local emergency department | 93.1 | 100 | Include |  |  |  |
| As far as possible, clinicians should recommend digital tools which allow users to input: contact details for their GP | 86.2 | 80 | Include |  |  |  |
| As far as possible, clinicians should recommend digital tools which allow users to input: contact details for other healthcare professionals involved in their care | 89.7 | 85 | Include |  |  |  |
| As far as possible, clinicians should recommend digital tools which allow users to input: contact details for face-to-face services and community supports in the young person’s local area | 93.1 | 95 | Include |  |  |  |
| As far as possible, clinicians should recommend digital tools which allow users to input: contact details for public hotlines for help in a crisis or an emergency | 93.1 | 100 | Include |  |  |  |
| As far as possible, clinicians should recommend digital tools which allow users to input: contact details for emergency contact/s | 96.6 | 95 | Include |  |  |  |
| As far as possible, clinicians should recommend digital tools which allow users to input: contact details for parent, carer or guardian/s (if different to emergency contacts) | 89.7 | 80 | Include |  |  |  |
| As far as possible, clinicians should recommend digital tools which allow users to input: contact details for other people (e.g., friends or family) who can be contacted for support | 89.7 | 80 | Include |  |  |  |
| As far as possible, clinicians should recommend digital tools which allow the treating clinician to view any data the young person enters in the digital tool relating to their suicidal thoughts or behaviours. | 37.9 | 40 | Exclude |  |  |  |
| As far as possible, clinicians should recommend digital tools which have the functionality to: display a summary of any mood tracking data (including data related to suicidal thoughts or behaviour) to the user | 82.8 | 70 | Re-rate | 80.8 | 73.7 | Exclude |
| As far as possible, clinicians should recommend digital tools which have the functionality to: send a summary of any mood tracking data (including data related to suicidal thoughts or behaviour) to the user | 72.4 | 70 | Re-rate | 73.1 | 68.4 | Exclude |
| As far as possible, clinicians should recommend digital tools which have the functionality to: automatically share data related to suicidal thoughts or behaviour with health professionals involved in the young person’s care | 44.8 | 45 | Exclude |  |  |  |
| As far as possible, clinicians should recommend digital tools which have the functionality to: enable synchronous conversation between the user and any mental health professionals | 48.3 | 40 | Exclude |  |  |  |
| As far as possible, clinicians should recommend digital tools which have the functionality to: enable direct contact between the young person and their treating mental health clinician (if applicable) | 86.2 | 45 | Re-rate | 76.9 | 52.6 | Exclude |
| As far as possible, clinicians should recommend digital tools which have the functionality to: notify emergency contacts that they have been nominated as such | 69.0 | 60 | Exclude |  |  |  |
| As far as possible, clinicians should recommend digital tools which have the functionality to: track the young person’s location (i.e., in case they need to be located in an emergency) | 41.4 | 40 | Exclude |  |  |  |
| As far as possible, clinicians should recommend digital tools which have the functionality to: automatically detect high suicide or self-harm risk or crisis situations (e.g., using data unobtrusively collected from smartphones and wearable devices) | 41.4 | 45 | Exclude |  |  |  |
| As far as possible, clinicians should recommend digital tools which, should the young person indicate via the tool that they are at imminent risk of harming themselves, have the functionality to: automatically contact emergency services | 55.2 | 85 | Re-rate | 65.4 | 78.9 | Exclude |
| 26. As far as possible, clinicians should recommend digital tools which, should the young person indicate via the tool that they are at imminent risk of harming themselves, have the functionality to: automatically notify the young person’s nominated emergency contacts or support people | 69.0 | 70 | Re-rate | 84.6 | 84.2 | Include |
| As far as possible, clinicians should recommend digital tools which, should the young person indicate via the tool that they are at imminent risk of harming themselves, have the functionality to: automatically notify the young person’s legal guardian (if applicable) | 44.8 | 65 | Exclude |  |  |  |
| As far as possible, clinicians should recommend digital tools which, should the young person indicate via the tool that they are at imminent risk of harming themselves, have the functionality to: automatically notify the young person’s primary treating mental health clinician | 62.1 | 70 | Re-rate | 80.8 | 68.4 | Exclude |
| As far as possible, clinicians should recommend digital tools which, should the young person indicate via the tool that they are at imminent risk of harming themselves, have the functionality to: automatically alert the duty worker at the young person’s mental health service (if applicable) | 58.6 | 55 | Exclude |  |  |  |
| As far as possible, clinicians should recommend digital tools which, should the young person indicate via the tool that they are at imminent risk of harming themselves, have the functionality to: prompt the user to contact emergency services | 96.6 | 85 | Include |  |  |  |
| As far as possible, clinicians should recommend digital tools which, should the young person indicate via the tool that they are at imminent risk of harming themselves, have the functionality to: prompt the user to notify their emergency contacts | 89.7 | 85 | Include |  |  |  |
| As far as possible, clinicians should recommend digital tools which, should the young person indicate via the tool that they are at imminent risk of harming themselves, have the functionality to: prompt the user to notify their primary treating mental health clinician | 82.8 | 80 | Include |  |  |  |
| As far as possible, clinicians should recommend digital tools which, should the young person indicate via the tool that they are at imminent risk of harming themselves, have the functionality to: prompt the user to alert the duty worker at the young person’s mental health service (if applicable) | 82.8 | 60 | Re-rate | 80.8 | 68.4 | Exclude |
| As far as possible, clinicians should recommend digital tools which, should the young person indicate via the tool that they are at imminent risk of harming themselves, have the functionality to: display the route to the nearest emergency department | 82.8 | 70 | Re-rate | 69.2 | 78.9 | Exclude |
| As far as possible, clinicians should recommend digital tools which, should the young person indicate via the tool that they are at imminent risk of harming themselves, have the functionality to: remind the young person to follow the steps on their safety plan |  |  | New in Round 2 | 100.0 | 100.0 | Include |
| As far as possible, clinicians should recommend digital tools which, should the young person indicate via the tool that they are experiencing a deterioration in mood, have the functionality to automatically push suggestions for coping strategies. |  |  | New in Round 2 | 96.2 | 94.7 | Include |
| Clinicians should not recommend digital tools which automatically share data with others (e.g., health professionals). |  |  | New in Round 2 | 23.1 | 42.1 | Exclude |
| Clinicians should only recommend digital tools which automatically share data with others if the young person provides appropriate informed consent for this. |  |  | New in Round 2 | 88.5 | 89.5 | Include |
| Clinicians should only recommend digital tools which automatically share data with others if there is sufficient infrastructure in place to respond in a timely way (e.g., in the case of an escalation in suicide risk being inputted into the tool and shared with a clinician). |  |  | New in Round 2 | 84.6 | 78.9 | Exclude |
| Clinicians should only recommend digital tools which automatically contact others (e.g., emergency services or family members) if the young person provides appropriate informed consent for this. |  |  | New in Round 2 | 84.6 | 84.2 | Include |
| As far as possible, clinicians should recommend digital tools to young people who experience suicidal thoughts or behaviour which are based on the following therapeutic approach/es: Cognitive Behavioural Therapy (CBT) | NA | 70 | Re-rate | NA | 73.7 | Exclude |
| As far as possible, clinicians should recommend digital tools to young people who experience suicidal thoughts or behaviour which are based on the following therapeutic approach/es: Dialectal Behavioural Therapy (DBT) | NA | 70 | Re-rate | NA | 73.7 | Exclude |
| As far as possible, clinicians should recommend digital tools to young people who experience suicidal thoughts or behaviour which are based on the following therapeutic approach/es: Interpersonal Psychotherapy | NA | 60 | Exclude |  |  |  |
| As far as possible, clinicians should recommend digital tools to young people who experience suicidal thoughts or behaviour which are based on the following therapeutic approach/es: Strengths-based/positive psychology approaches | NA | 70 | Re-rate | NA | 68.4 | Exclude |
| As far as possible, clinicians should recommend digital tools to young people who experience suicidal thoughts or behaviour which are based on the following therapeutic approach/es: Motivational interviewing | NA | 70 | Re-rate | NA | 52.6 | Exclude |
| As far as possible, clinicians should recommend digital tools to young people who experience suicidal thoughts or behaviour which are based on the therapeutic approach/es most suited to the young person’s clinical presentation and symptoms (e.g., a DBT-based app if the young person struggles with distress tolerance and emotion regulation). |  |  | New in Round 2 | NA | 94.7 | Include |
| Clinicians should assess: the young person’s use of digital tools that are specifically used for the purpose of managing their suicidal thoughts or behaviour (including social media platforms or pages) | 93.1 | 65 | Re-rate | 92.3 | 78.9 | Exclude |
| Clinicians should assess: the young person’s use of digital tools which may impact on their suicidal thoughts or behaviour (positively or negatively), even if not specifically used for this purpose (e.g., social media platforms or pages) | 86.2 | 75 | Re-rate | 84.6 | 68.4 | Exclude |
| Clinicians should only recommend new digital tools if they meet a need that cannot already be met by other digital tools or technology already used by the young person. |  |  | New in Round 2 | 57.7 | 63.2 | Exclude |
| Clinicians should educate young people about how to use digital tools safely (e.g., take breaks from social media or other digital-based platforms if needed for their mental health, muting certain notifications, limiting time on other apps, utilising focus modes, blocking certain people, etc.). |  |  | New in Round 2 | 96.2 | 94.7 | Include |
| Clinicians should encourage open and ongoing discussions about the young person's use of digital tools and technology, including social media. |  |  | New in Round 2 | 88.5 | 84.2 | Include |
| Clinicians should assess the use of digital tools which may impact on suicidal thoughts or behaviour: at the start of the young person’s episode of care | 86.2 | 80 | Include |  |  |  |
| Clinicians should assess the use of digital tools which may impact on suicidal thoughts or behaviour: periodically throughout the young person’s episode of care (if applicable) | 93.1 | 90 | Include |  |  |  |
| If a young person uses digital tools that may impact their suicidal thoughts or behaviour, clinicians should: work collaboratively with the young person to evaluate the risks and benefits |  |  | New in Round 2 | 96.2 | 100.0 | Include |
| If a young person uses digital tools that may impact their suicidal thoughts or behaviour, clinicians should: empower the young person to evaluate whether their use of digital tools/technology is helpful or harmful |  |  | New in Round 2 | 96.2 | 94.7 | Include |
| If a young person uses digital tools that may impact their suicidal thoughts or behaviour, clinicians should: empower the young person to manage their use of digital tools/technology to maximise efficacy and safety |  |  | New in Round 2 | 96.2 | 94.7 | Include |
| If a young person is using a digital tool that may impact their suicidal thoughts or behaviour that the clinician is not familiar with, the clinician should examine: the quality of the tool (e.g., whether it is evidence-based) | 96.6 | 80 | Include |  |  |  |
| If a young person is using a digital tool that may impact their suicidal thoughts or behaviour that the clinician is not familiar with, the clinician should examine: whether or not the tool contains potentially harmful content [as rated in Section 1, Q5] | 96.6 | 90 | Include |  |  |  |
| If a young person uses a digital tool that may impact their suicidal thoughts or behaviour, and the clinician is concerned that these are of poor quality and/or contain potentially harmful content [as rated in Section 1, Q5], the clinician should: direct the young person to more appropriate digital tools or resources | 93.1 | 95 | Include |  |  |  |
| If a young person uses a digital tool that may impact their suicidal thoughts or behaviour, and the clinician is concerned that these are of poor quality and/or contain potentially harmful content [as rated in Section 1, Q5], the clinician should: provide psychoeducation about how to identify safer digital tools or resources | 93.1 | 100 | Include |  |  |  |
| If a young person uses a digital tool that may impact their suicidal thoughts or behaviour, and the clinician is concerned that these are of poor quality and/or contain potentially harmful content [as rated in Section 1, Q5], the clinician should: address any safety issues (e.g., exposure to potentially harmful content) collaboratively with the young person | 96.6 | 100 | Include |  |  |  |
| Clinicians should educate young people about how they can use their existing social media platforms (e.g., Facebook, Instagram, TikTok, etc.) as safely and beneficially as possible. | 93.1 | 80 | Include |  |  |  |
| Clinicians should avoid introducing new digital tools to the young person, and instead utilise the digital tools the young person already uses (including social media platforms). | 20.7 | 15 | Exclude |  |  |  |
| When proposing use of a digital tool to a young person, clinicians should discuss or explain: the evidence supporting the digital tool for suicidal thoughts or behaviour | 75.9 | 80 | Re-rate | 76.9 | 73.7 | Exclude |
| When proposing use of a digital tool to a young person, clinicians should discuss or explain: the role of the tool in supporting the young person’s suicidal thoughts or behaviour (e.g., whether it is designed for distraction, for accessing help, or some other reason) | 100.0 | 100 | Include |  |  |  |
| When proposing use of a digital tool to a young person, clinicians should discuss or explain: whether and how the young person should use the tool if they think they might engage in suicidal behaviour | 96.6 | 95 | Include |  |  |  |
| When proposing use of a digital tool to a young person, clinicians should discuss or explain: any potential negative impacts of the tool on the young person’s suicidal thoughts or behaviour | 89.7 | 90 | Include |  |  |  |
| When proposing use of a digital tool to a young person, clinicians should discuss or explain: what the young person should do if they experience any negative impacts of the tool on their suicidal thoughts or behaviour | 100.0 | 100 | Include |  |  |  |
| When proposing use of a digital tool to a young person, clinicians should discuss or explain: any safety procedures involved in using the tool, including an explanation of what will happen if the young person reports that their suicidal thoughts or behaviour are escalating [please note, you will be asked to rate safety procedures in a later section of the survey] | 96.6 | 100 | Include |  |  |  |
| When proposing use of a digital tool to a young person, clinicians should discuss or explain: what will happen if it becomes apparent that the digital tool is unsuitable, unsafe, or ineffective for the young person | 96.6 | 90 | Include |  |  |  |
| When proposing use of a digital tool to a young person, clinicians should discuss or explain: what will happen to the young person’s data (e.g., whether and what information inputted into the tool will be documented on their electronic medical record) | 96.6 | 95 | Include |  |  |  |
| When proposing use of a digital tool to a young person, clinicians should discuss or explain: that the tool might be helpful for some people, but unhelpful or triggering for others (if applicable) |  |  | New in Round 2 | 100.0 | 100.0 | Include |
| When first introducing a digital tool to a young person, clinicians should: complete the sign-up process in-session |  |  | New in Round 2 | 73.1 | 36.8 | Exclude |
| When first introducing a digital tool to a young person, clinicians should: demonstrate how to use it in-session |  |  | New in Round 2 | 92.3 | 73.7 | Exclude |
| When first introducing a digital tool to a young person, clinicians should: - check the young person’s understanding of and confidence using the tool, and allow additional time to explain/demonstrate it if needed |  |  | New in Round 2 | 96.2 | 89.5 | Include |
| When first introducing a digital tool to a young person, clinicians should: explain how the tool's safety, acceptability, and efficacy for the young person will be reviewed |  |  | New in Round 2 | 96.2 | 94.7 | Include |
| When proposing the use of a digital tool to a young person, clinicians should provide a range of options for the young person to choose from. |  |  | New in Round 2 | 76.9 | 36.8 | Exclude |
| If the clinician believes there is any possibility the tool might have a negative impact on the young person, the clinician should: suggest alternative digital tools the young person can use if they experience a negative impact |  |  | New in Round 2 | 88.5 | 73.7 | Exclude |
| If the clinician believes there is any possibility the tool might have a negative impact on the young person, the clinician should: suggest non-digital alternatives the young person can use if they experience a negative impact |  |  | New in Round 2 | 80.8 | 84.2 | Include |
| Clinicians should contact the young person's parent, carer, or guardian/s (where appropriate) to: inform them that they have asked the young person to use a digital tool which is designed to support them with their suicidal thoughts or behaviour | 55.2 | 55 | Exclude |  |  |  |
| Clinicians should contact the young person's parent, carer, or guardian/s (where appropriate) to: explain how the young person can, or should, use the digital tool when experiencing suicidal thoughts or behaviour | 65.5 | 65 | Exclude |  |  |  |
| Clinicians should contact the young person's parent, carer, or guardian/s (where appropriate) to: discuss the parent, carer, or guardian/s role in any risk escalation process (e.g., whether they might be contacted if the clinician is concerned about the young person’s safety) | 72.4 | 75 | Re-rate | 84.6 | 84.2 | Include |
| Clinicians should contact the young person's parent, carer, or guardian/s (where appropriate) to: explore any concerns they may have around the use of technology in general, or with the specific digital tool recommended | 75.9 | 65 | Re-rate | 96.2 | 78.9 | Exclude |
| Clinicians should discuss with the young person any plans to talk to their parent, carer, or guardian/s regarding their use of the digital tool and aim to respect the young person’s wishes regarding what information is shared with them. |  |  | New in Round 2 | 88.5 | 100.0 | Include |
| Clinicians should only inform the young person’s parent, carer, or guardian/s about their use of the digital tool if this: would be standard practice based on the young person’s age and developmental stage |  |  | New in Round 2 | 80.8 | 94.7 | Include |
| Clinicians should only inform the young person’s parent, carer, or guardian/s about their use of the digital tool if this: is not likely to increase risk or distress for the young person |  |  | New in Round 2 | 96.2 | 68.4 | Exclude |
| Clinicians should only inform the young person’s parent, carer, or guardian/s about their use of the digital tool if this: is not likely to impede the young person’s ability to use the digital tool |  |  | New in Round 2 | 84.6 | 68.4 | Exclude |
| Clinicians should establish or confirm with the young person: the processes for communication about suicidal thoughts or behaviour | 96.6 | 100 | Include |  |  |  |
| Clinicians should establish or confirm with the young person: whether the young person can contact them via digital means to talk about their suicidal thoughts or behaviour | 100.0 | 95 | Include |  |  |  |
| Clinicians should establish or confirm with the young person: how the young person can contact them via digital means to talk about their suicidal thoughts or behaviour (e.g., email, text message) | 100.0 | 95 | Include |  |  |  |
| Clinicians should establish or confirm with the young person: when the young person can contact them via digital means to talk about their suicidal thoughts or behaviour (e.g., time of day, days of the week) | 100.0 | 90 | Include |  |  |  |
| Clinicians should establish or confirm with the young person: the approximate length of time to expect a response back from the clinician | 93.1 | 100 | Include |  |  |  |
| Clinicians should establish or confirm with the young person: the approximate length of time for the clinician to expect a response back from the young person | 75.9 | 80 | Re-rate | 92.3 | 94.7 | Include |
| Clinicians should establish or confirm with the young person: what the delay in response is likely to be outside the clinician’s working hours | 100.0 | 95 | Include |  |  |  |
| Clinicians should establish or confirm with the young person: whether or not the young person can contact the clinician via digital means if they are feeling unable to keep themselves safe | 100.0 | 100 | Include |  |  |  |
| Clinicians should establish or confirm with the young person: alternative avenues for accessing crisis support if they are unable to reach the clinician (e.g., help lines) | 100.0 | 100 | Include |  |  |  |
| Clinicians should establish or confirm with the young person: processes to be followed if the young person loses their device or runs out of battery (e.g., to let the clinician know, so they don’t worry about their safety) | 89.7 | 85 | Include |  |  |  |
| Clinicians should establish or confirm with the young person: any processes to be followed if the clinician does not receive a response from the young person within the expected time frame (e.g., calling an emergency contact) | 100.0 | 95 | Include |  |  |  |
| Clinicians should establish or confirm with the young person: any processes to be followed in the case of a technological failure | 82.8 | 90 | Include |  |  |  |
| Clinicians should periodically remind the young person of the agreed-on processes for digital communication about suicidal thoughts and behaviour. | 69.0 | 90 | Re-rate | 80.8 | 84.2 | Include |
| Clinicians should provide a copy of the agreed-upon processes for digital communication to the young person (e.g., written/typed format). |  |  | New in Round 2 | 84.6 | 73.7 | Exclude |
| Clinicians should not set up the expectation that they will review or respond to digital communication between sessions, unless this is part of the service model. |  |  | New in Round 2 | 65.4 | 78.9 | Exclude |
| Clinicians should not set up the expectation that they will review or respond to digital communication between sessions, unless there is an acute clinical treatment plan in place (i.e., this is part of the young person’s treatment plan due to their level of suicide risk). |  |  | New in Round 2 | 61.5 | 84.2 | Exclude |
| Where possible, if clinicians communicate using a digital tool, this should allow for read/received receipts. | 79.3 | 60 | Re-rate | 26.9 | 52.6 | Exclude |
| Clinicians should avoid using read/received receipts, as it may be distressing for a young person to know the clinician has read, but not responded to, their message. |  |  | New in Round 2 | 65.4 | 36.8 | Exclude |
| If possible, clinicians should turn read/received receipts on. | 65.5 | 45 | Exclude |  |  |  |
| If possible, clinicians should request the young person turn read/received receipts on. | 75.9 | 60 | Re-rate | 57.7 | 52.6 | Exclude |
| Clinicians and/or services, as appropriate, should develop policies and procedures for identifying and responding to suicidal thoughts or behaviour conveyed via digital tools or technology. | NA | 95 | Include |  |  |  |
| Any policies and procedures for identifying and responding to suicidal thoughts or behaviour in a digital context should Include: designation of roles and responsibilities (e.g., who contacts emergency services) | NA | 100 | Include |  |  |  |
| Any policies and procedures for identifying and responding to suicidal thoughts or behaviour in a digital context should Include: specifications for when, how, and by whom suicidal thoughts and behaviour communicated via, or inputted into, a digital tool will be monitored | NA | 100 | Include |  |  |  |
| Any policies and procedures for identifying and responding to suicidal thoughts or behaviour in a digital context should Include: clear processes for managing escalation of suicide risk, which specify who is accountable at what stage of the escalation process | NA | 100 | Include |  |  |  |
| Any policies and procedures for identifying and responding to suicidal thoughts or behaviour in a digital context should Include: a commitment to working collaboratively with the young person | NA | 95 | Include |  |  |  |
| Any policies and procedures for identifying and responding to suicidal thoughts or behaviour in a digital context should Include: a commitment to allowing the young person “dignity of risk” (the right of individuals to choose to take some risk in engaging in life experiences) | NA | 80 | Include |  |  |  |
| Monitoring digital tools for suicidal thoughts and behaviour should occur with sufficient time to respond if required (e.g., monitored within GP practice hours, if contacting the GP is part of risk escalation procedures). | NA | 90 | Include |  |  |  |
| Clinicians and/or services, as appropriate, should clearly communicate to young people whether, and how often, information about suicidal thoughts and behaviour inputted into the digital tool will be monitored. | 89.7 | 95 | Include |  |  |  |
| Where digital tools are used to monitor suicidal thoughts and behaviour in between therapy sessions, clinicians should review the results of the monitoring: at each therapy session | 82.8 | 80 | Include |  |  |  |
| Where digital tools are used to monitor suicidal thoughts and behaviour in between therapy sessions, clinicians should review the results of the monitoring: at least once in between therapy sessions | 72.4 | 45 | Re-rate | 73.1 | 31.6 | Exclude |
| Where digital tools are used to monitor suicidal thoughts and behaviour in between therapy sessions, clinicians should review the results of the monitoring: every day in between therapy sessions | 6.9 | 5 | Exclude |  |  |  |
| Where digital tools are used to monitor suicidal thoughts and behaviour in between therapy sessions, clinicians should only review the results of the monitoring between sessions if they have the capacity and resources to respond in the case of escalating suicidal thoughts or behaviour. |  |  |  | NA | 63.2 | Exclude |
| Clinicians should work with the young person to develop a safety plan specific to digital tools, in addition to any standard safety plan. | 93.1 | 85 | Include |  |  |  |
| Any safety plan specific to digital tools should Include the following: coping strategies that involve the use of digital tools or technology | 89.7 | 80 | Include |  |  |  |
| Any safety plan specific to digital tools should Include the following: instructions on how to use digital tools or technology to seek informal support in a crisis | 96.6 | 90 | Include |  |  |  |
| Any safety plan specific to digital tools should Include the following: instructions on how to use digital tools or technology to seek professional help | 96.6 | 90 | Include |  |  |  |
| Any safety plan specific to digital tools should Include the following: details of how the safety, acceptability and efficacy of digital tools will be monitored |  |  | New in Round 2 | 88.5 | 68.4 | Exclude |
| If a young person has a safety plan, clinicians should ensure it is available in a digital format (e.g., through a safety planning app or a PDF sent to their email address). |  |  | New in Round 2 | 88.5 | 73.7 | Exclude |
| Clinicians should integrate safety planning related to digital tools into the young person's standard safety plan, rather than creating a separate safety plan for digital tools. |  |  | New in Round 2 | 88.5 | 84.2 | Include |
| If a young person has a safety plan, clinicians should ensure it is available in a digital format (e.g., through a safety planning app). | 89.7 | 70 | Re-rate | 84.6 | 73.7 | Exclude |
| Clinicians should develop an individualised "emergency response plan" for every young person they work with, detailing the steps to be taken by the clinician if they are worried about the young person based on information shared through a digital tool | 100.0 | 70 | Re-rate | 96.2 | 89.5 | Include |
| Any "emergency response plan" should Include: the young person’s home address | 96.6 | 80 | Include |  |  |  |
| Any "emergency response plan" should Include: other locations the young person commonly visits (e.g., other family member’s home, or school) | 79.3 | 65 | Re-rate | 84.6 | 52.6 | Exclude |
| Any "emergency response plan" should Include: warning signs that may indicate a young person’s suicidal thoughts or behaviour are escalating | 96.6 | 90 | Include |  |  |  |
| Any "emergency response plan" should Include: contact information for the young person’s chosen support people (e.g., family, friends) | 96.6 | 95 | Include |  |  |  |
| Any "emergency response plan" should Include: contact information for the young person’s legal guardian/s | 86.2 | 85 | Include |  |  |  |
| Any "emergency response plan" should Include: contact information for other professionals involved in the young person’s care | 96.6 | 85 | Include |  |  |  |
| Any "emergency response plan" should Include: details of the young person’s local emergency department | 89.7 | 80 | Include |  |  |  |
| Any "emergency response plan" should Include: details of how the extent of suicidal thoughts or behaviour will be assessed if the young person can be contacted (including who will be contacted and in what circumstances) | 93.1 | 75 | Re-rate | 100.0 | 89.5 | Include |
| Any "emergency response plan" should Include: details of how the extent of suicidal thoughts or behaviour will be assessed if the young person cannot be contacted (including who will be contacted and in what circumstances) | 89.7 | 75 | Re-rate | 100.0 | 94.7 | Include |
| Any "emergency response plan" should Include: details of procedures to be followed by the clinician and/or services in the event that the young person is known or suspected to be at imminent risk of harming themselves (e.g., about to engage in potentially life-threatening suicidal behaviour) | 93.1 | 95 | Include |  |  |  |
| Any "emergency response plan" should Include: details of any emergency procedures specific to the location or setting in which the young person might use the digital tool (e.g., at school) | 86.2 | 85 | Include |  |  |  |
| Where possible, clinicians should liaise with the relevant staff at locations where the young person may access the digital tool (e.g., at school) to establish and maintain emergency response processes across settings. | 69.0 | 60 | Exclude |  |  |  |
| Before proposing a digital tool to a young person, clinicians should conduct a thorough suicide and self-harm risk assessment, including an assessment of the factors that may increase the chances that the young person will engage in suicidal behaviour | 96.6 | 100 | Include |  |  |  |
| For any digital tools which allow the young person to input a rating of their suicidal thoughts, clinicians should work collaboratively with the young person to determine what each rating on the scale represents (e.g., on a scale of 1-10, 7 or above represents ‘I am worried I might not be able to keep myself safe’, 9 represents ‘I have a plan and intend to attempt suicide’). | 96.6 | 90 | Include |  |  |  |
| Clinicians should request permission from young people to prospectively view any of their publicly available social media profiles or posts, if this would assist in assessing their risk of suicide or self-harm at any time during their episode of care. | 55.2 | 30 | Exclude |  |  |  |
| If the clinician becomes concerned that the young person might engage in suicidal behaviour through information inputted into a digital tool or conveyed digitally, they should attempt to contact the young person as soon as possible to assess their likelihood of engaging in suicidal behaviour. | 82.8 | 90 | Include |  |  |  |
| If the clinician attempts to contact the young person to assess their likelihood of engaging in suicidal behaviour, and is not able to reach them, they should: check the young person’s social media profiles to see if they have posted anything that might help with the assessment of risk | 31.0 | 10 | Exclude |  |  |  |
| If the clinician attempts to contact the young person to assess their likelihood of engaging in suicidal behaviour, and is not able to reach them, they should: check the young person’s social media profiles to see if they have posted anything that might help with the assessment of risk, only if permission has been obtained by the young person | 58.6 | 20 | Exclude |  |  |  |
| If the clinician attempts to contact the young person to assess their likelihood of engaging in suicidal behaviour, and is not able to reach them, they should: follow the processes of any previously established emergency response plan (e.g., contact the emergency contact, if this is part of the plan) | 93.1 | 90 | Include |  |  |  |
| If the clinician becomes concerned that the young person is at imminent risk of harming themselves based on information inputted into the digital tool or conveyed digitally, the clinician should: confirm the young person’s current location, if possible | 79.3 | 85 | Re-rate | 92.3 | 84.2 | Include |
| If the clinician becomes concerned that the young person is at imminent risk of harming themselves based on information inputted into the digital tool or conveyed digitally, the clinician should: follow the processes of any previously established emergency response plan | 96.6 | 95 | Include |  |  |  |
| Clinicians should only recommend or endorse online communities to young people who experience suicidal thoughts or behaviour which: have a clear purpose | 86.2 | 70 | Re-rate | 88.5 | 89.5 | Include |
| Clinicians should only recommend or endorse online communities to young people who experience suicidal thoughts or behaviour which: have clear eligibility criteria | 82.8 | 60 | Re-rate | 80.8 | 78.9 | Exclude |
| Clinicians should only recommend or endorse online communities to young people who experience suicidal thoughts or behaviour which: have clear terms of use | 100.0 | 80 | Include |  |  |  |
| Clinicians should only recommend or endorse online communities to young people who experience suicidal thoughts or behaviour which: are moderated | 79.3 | 80 | Re-rate | 88.5 | 89.5 | Include |
| Clinicians should only recommend or endorse online communities to young people who experience suicidal thoughts or behaviour which: are moderated by trained professionals | 96.6 | 75 | Re-rate | 96.2 | 89.5 | Include |
| Clinicians should only recommend or endorse online communities to young people who experience suicidal thoughts or behaviour which: are moderated by peers with lived experience | 58.6 | 30 | Exclude |  |  |  |
| Clinicians should only recommend or endorse online communities to young people who experience suicidal thoughts or behaviour which: clearly display details of when and how often the community is moderated | 96.6 | 60 | Re-rate | 88.5 | 94.7 | Include |
| Clinicians should only recommend or endorse online communities to young people who experience suicidal thoughts or behaviour which: clearly display conditions under which reporting posts or users is appropriate | 96.6 | 80 | Include |  |  |  |
| Clinicians should only recommend or endorse online communities to young people who experience suicidal thoughts or behaviour which: clearly display information about crisis supports | 96.6 | 85 | Include |  |  |  |
| Clinicians should only recommend or endorse online communities to young people who experience suicidal thoughts or behaviour which: outline any limits to confidentiality | 100.0 | 90 | Include |  |  |  |
| Clinicians should only recommend or endorse online communities to young people who experience suicidal thoughts or behaviour which: are co-designed with young people with lived experience of suicidal thoughts or behaviour | 93.1 | 55 | Re-rate | 92.3 | 63.2 | Exclude |
| Clinicians should only recommend or endorse online communities to young people who experience suicidal thoughts or behaviour which: allow users to report posts | 100.0 | 60 | Re-rate | 96.2 | 73.7 | Exclude |
| Clinicians should only recommend or endorse online communities to young people who experience suicidal thoughts or behaviour which: allow users to report other users | 86.2 | 55 | Re-rate | 92.3 | 68.4 | Exclude |
| Clinicians should only recommend or endorse online communities to young people who experience suicidal thoughts or behaviour which: allow users to participate using a pseudonym | 75.9 | 30 | Re-rate | 84.6 | 31.6 | Exclude |
| Clinicians should only recommend or endorse online communities to young people who experience suicidal thoughts or behaviour which: allow private messaging between users and any moderators | 72.4 | 50 | Re-rate | 69.2 | 36.8 | Exclude |
| Clinicians should only recommend or endorse online communities to young people who experience suicidal thoughts or behaviour which: allow private messaging between users | 6.9 | 0 | Exclude |  |  |  |
| Clinicians should only recommend or endorse online communities to young people who experience suicidal thoughts or behaviour which: allow private messaging between users, only if these can be viewed by any moderators | 62.1 | 40 | Exclude |  |  |  |
| Clinicians should only recommend or endorse online communities to young people who experience suicidal thoughts or behaviour which: are available for users to access 24 hours a day | 89.7 | 55 | Re-rate | 84.6 | 52.6 | Exclude |
| Clinicians should only recommend or endorse online communities to young people who experience suicidal thoughts or behaviour which: are available for users to access only during certain hours (e.g., business hours) | 13.8 | 10 | Exclude |  |  |  |
| Clinicians should only recommend or endorse online communities to young people who experience suicidal thoughts or behaviour which: Include a message that normalises the experience of suicidal thoughts |  |  | New in Round 2 | 50.0 | 42.1 | Exclude |
| Clinicians should only recommend or endorse online communities to young people who experience suicidal thoughts or behaviour which: are available for users to access only while being actively moderated (e.g., accessible 24 hours a day, only if the platform is moderated for 24 hours a day) |  |  | New in Round 2 | 80.8 | 68.4 | Exclude |
| Clinicians should only recommend or endorse online communities to young people who experience suicidal thoughts or behaviour which: allow for anonymous participation |  |  | New in Round 2 | 42.3 | 5.3 | Exclude |
| Clinicians should only recommend or endorse online communities to young people who experience suicidal thoughts or behaviour which: prohibit anonymous participation |  |  | New in Round 2 | 23.1 | 36.8 | Exclude |
| Before recommending an online community to a young person, clinicians should ensure the young person understands any terms of use associated with it. | 96.6 | 90 | Include |  |  |  |
| Clinicians should not recommend or endorse online communities to young people who experience suicidal thoughts or behaviour, unless they are very familiar with the online community. |  |  | New in Round 2 | 73.1 | 94.7 | Exclude |
| If recommending any online communities to young people who experience suicidal thoughts or behaviour, clinicians should discuss with the young person: the risks and benefits of online communities |  |  | New in Round 2 | 96.2 | 94.7 | Include |
| If recommending any online communities to young people who experience suicidal thoughts or behaviour, clinicians should discuss with the young person: how to communicate safely online about suicide (e.g., drawing on the #chatsafe guidelines) |  |  | New in Round 2 | 100.0 | 94.7 | Include |
| If recommending any online communities to young people who experience suicidal thoughts or behaviour, clinicians should discuss with the young person: what they should do if they need support during or after accessing the online community |  |  | New in Round 2 | 96.2 | 94.7 | Include |
| After recommending any online communities to young people who experience suicidal thoughts or behaviour, the clinician should regularly check in with the young person to ensure that: they are comfortable in the online space |  |  | New in Round 2 | 100.0 | 73.7 | Exclude |
| After recommending any online communities to young people who experience suicidal thoughts or behaviour, the clinician should regularly check in with the young person to ensure that: they are communicating safely in the online space |  |  | New in Round 2 | 96.2 | 63.2 | Exclude |
| After recommending any online communities to young people who experience suicidal thoughts or behaviour, the clinician should regularly check in with the young person to ensure that: the online community is not associated with distress or increased likelihood of suicidal thoughts or behaviour |  |  | New in Round 2 | 100.0 | 89.5 | Include |
| Clinicians should educate and empower young people to: recognise which online communities may be helpful or harmful to them |  |  | New in Round 2 | 100.0 | 84.2 | Include |
| Clinicians should educate and empower young people to: make informed decisions about which online communities to use |  |  | New in Round 2 | 96.2 | 84.2 | Include |
| Clinicians should only recommend online communities that are moderated by peer workers (i.e., with lived experience), if it can be confirmed that the peer moderators have had: appropriate training |  |  | New in Round 2 | 96.2 | 68.4 | Exclude |
| Clinicians should only recommend online communities that are moderated by peer workers (i.e., with lived experience), if it can be confirmed that the peer moderators have had: ongoing supervision and support |  |  | New in Round 2 | 96.2 | 73.7 | Exclude |
| Clinicians should document: all conversations they have with the young person about digital tools | NA | 85 | Include |  |  |  |
| Clinicians should document: any agreed-upon conditions of using a digital tool | NA | 90 | Include |  |  |  |
| Clinicians should document: any agreed-upon processes for communicating digitally about suicidal thoughts or behaviours | NA | 95 | Include |  |  |  |
| Clinicians should document: all data inputted into the digital tool by the young person | NA | 15 | Exclude |  |  |  |
| Clinicians should document: only data inputted into the digital tool by the young person which specifically relates to their suicidal thoughts or behaviour | NA | 55 | Exclude |  |  |  |
| Clinicians should document: any steps taken in response to data inputted by the young person regarding their suicidal thoughts or behaviour (e.g., whether emergency response plans were activated) | NA | 100 | Include |  |  |  |
| Clinicians should document: any media (e.g., images, audio files) shared by the young person through the digital tool | NA | 20 | Exclude |  |  |  |
| Clinicians should document: only media (e.g., images, audio files) shared by the young person which specifically relates to their suicidal thoughts or behaviour | NA | 35 | Exclude |  |  |  |
| Services should develop a "digital strategy" which outlines how digital tools and technology should and should not be used with young people who experience suicidal thoughts or behaviour as part of their service provision. | NA | 95 | Include |  |  |  |
| Any digital strategy should be co-designed with: young people who access the service | NA | 80 | Include |  |  |  |
| Any digital strategy should be co-designed with: young people with lived experience of suicidal thoughts or behaviour | NA | 70 | Re-rate | NA | 84.2 | Include |
| Any digital strategy should be co-designed with: staff who work at the service | NA | 85 | Include |  |  |  |
| Services wanting to integrate digital tools and technology into standard care for young people experiencing suicidal thoughts and behaviours should provide staff training on: how to incorporate digital tools into their standard care delivery | NA | 100 | Include |  |  |  |
| Services wanting to integrate digital tools and technology into standard care for young people experiencing suicidal thoughts and behaviours should provide staff training on: how to use any specific individual tools encouraged or required to be used in the service | NA | 100 | Include |  |  |  |
| Services wanting to integrate digital tools and technology into standard care for young people experiencing suicidal thoughts and behaviours should provide staff training on: documentation requirements, including what data are considered to be part of a medical record and/or transferred to the young person’s medical record | NA | 95 | Include |  |  |  |
| Services wanting to integrate digital tools and technology into standard care for young people experiencing suicidal thoughts and behaviours should provide staff training on: secure management of data | NA | 100 | Include |  |  |  |
| Services wanting to integrate digital tools and technology into standard care for young people experiencing suicidal thoughts and behaviours should provide staff training on: evaluation of the appropriateness, quality, safety and efficacy of digital tools | NA | 95 | Include |  |  |  |
| Services wanting to integrate digital tools and technology into standard care for young people experiencing suicidal thoughts and behaviours should provide or obtain the following resources: secure data storage/security measures | NA | 90 | Include |  |  |  |
| Services wanting to integrate digital tools and technology into standard care for young people experiencing suicidal thoughts and behaviours should provide or obtain the following resources: necessary technology (including mobile data) to clinicians | NA | 90 | Include |  |  |  |
| Services wanting to integrate digital tools and technology into standard care for young people experiencing suicidal thoughts and behaviours should provide or obtain the following resources: necessary technology (including mobile data) to young people | NA | 75 | Re-rate | NA | 57.9 | Exclude |
| Services wanting to integrate digital tools and technology into standard care for young people experiencing suicidal thoughts and behaviours should provide or obtain the following resources: organisational subscriptions to tools with subscription costs | NA | 65 | Exclude |  |  |  |
| Services wanting to integrate digital tools and technology into standard care for young people experiencing suicidal thoughts and behaviours should provide or obtain the following resources: access to a range of different digital tools to meet the needs of different users | NA | 80 | Include |  |  |  |
| Services wanting to integrate digital tools and technology into standard care for young people experiencing suicidal thoughts and behaviours should: incentivise clinicians to use digital tool/s | NA | 30 | Exclude |  |  |  |
| Services wanting to integrate digital tools and technology into standard care for young people experiencing suicidal thoughts and behaviours should: actively promote recommended digital tools to staff | NA | 80 | Include |  |  |  |
| Services should develop transparent governance processes for digital tools which stipulate: privacy and security processes (including standards for data storage, use and sharing) | NA | 100 | Include |  |  |  |
| Services should develop transparent governance processes for digital tools which stipulate: safety measures | NA | 100 | Include |  |  |  |
| Services should develop transparent governance processes for digital tools which stipulate: how feasibility of the digital tool/s will be evaluated | NA | 80 | Include |  |  |  |
| Services should develop transparent governance processes for digital tools which stipulate: how acceptability of the digital tool/s will be evaluated | NA | 80 | Include |  |  |  |
| Services should develop: procedures to identify and address equity concerns (e.g., where young people might be unable to use digital tools due to limited access to data or devices) | NA | 95 | Include |  |  |  |
| Services should develop: impact assessment processes to assess the potential impact of proposed digital tools | NA | 80 | Include |  |  |  |
| Services should develop: impact assessment processes to assess the impact of digital tools during implementation | NA | 85 | Include |  |  |  |
| Services should: ensure data storage, use and sharing policies are transparent to young people | NA | 95 | Include |  |  |  |
| Services should: ensure that, as far as possible, young people can control what data can be shared and with whom | NA | 85 | Include |  |  |  |
| Services should: plan for appropriate organisational responses in the event that digital tools cause harm (e.g., security breach, data leaks), with clear designation of roles and duties (e.g., informing service users of a potential data breach) | NA | 100 | Include |  |  |  |
| Services should: clarify details of security measures, such as who will be responsible for implementing them and monitoring compliance | NA | 100 | Include |  |  |  |
| Services should: take reasonable steps to ensure that the young people who choose not to engage with digital tools retain a high quality of service provision and are not disadvantaged for their choice | NA | 95 | Include |  |  |  |
| If the service has developed its own digital tool, including an online community, they should ensure this complies with the service's existing governance structures. | NA | 100 | Include |  |  |  |
| Clinicians and services wanting to use digital tools as part of a young person’s treatment plan should ensure that there is no cost to the user. |  |  | New in Round 2 | NA | 52.6 | Exclude |
